# Severity-dependent IgG epitope profiling in COVID-19 reveals differential recognition of pathogen-derived antigens

**DOI:** 10.3389/fimmu.2025.1668223

**Published:** 2025-09-03

**Authors:** Lais Alves do Nascimento, NicolleRakanidis Machado, João Vitor da Silva Borges, Beatriz Oliveira Fagundes, Isabella Siuffi Bergamasco, Fabio da Ressureição Sgnotto, André Luis Lacerda Bachi, Maria Notomi Sato, Jefferson Russo Victor

**Affiliations:** ^1^ Laboratory of Medical Investigation LIM-56, Division of Dermatology, Medical School, University of São Paulo, São Paulo, Brazil; ^2^ School of Medicine, Santo Amaro University (UNISA), São Paulo, Brazil; ^3^ PostGraduation Program in Health Sciences, Santo Amaro University (UNISA), São Paulo, Brazil

**Keywords:** COVID-19, IgG, antibody repertoire, peptide microarray, pathogen epitopes, immune modulation, cross-reactivity, autoantibodies

## Abstract

**Background:**

The contribution of antibody-mediated responses to COVID - 19 outcomes remains unclear, particularly regarding cross-reactivity with unrelated pathogens. While co-infections are known to influence disease progression, the broader landscape of IgG reactivity during SARS-CoV-2 infection has not been systematically explored.

**Methods:**

We employed a high-density peptide microarray containing 4,344 linear epitopes from 37 viruses, 27 bacteria, 17 parasites, and 8 fungi to characterize serum IgG repertoires from individuals with moderate (n = 39) or severe (n = 40) COVID - 19. Controls included pre-pandemic healthy donors and a pooled intravenous immunoglobulin (IVIg) formulation. Data analysis included intensity ranking, epitope mapping, and comparative analysis of mean signal intensities for each epitope between the COVID-Mod and COVID-Sev groups.

**Results:**

COVID - 19 patients showed widespread IgG reactivity against diverse pathogens, with patterns differing by disease severity. Severe cases displayed broader and more intense reactivity, notably against hepatitis C virus (HCV), SARS-CoV-1, influenza A, Mycobacterium tuberculosis, and Plasmodium falciparum. Moderate cases showed preferential recognition of epitopes from HTLV-I, Neisseria meningitidis, and Trypanosoma cruzi. These findings suggest that SARS-CoV-2 infection modulates pre-existing humoral memory, possibly through epitope spreading or immune reprogramming.

**Conclusions:**

SARS-CoV-2 infection reshapes the IgG epitope repertoire in a severity-dependent manner, extending to antigens from unrelated pathogens. This phenomenon may reflect underlying immune dysregulation or idiotype-driven interactions. Comprehensive profiling of pathogen-related IgG responses may reveal potential biomarkers of disease severity. This phenomenon may inform future investigations aimed at improving personalized management strategies for co-infected or immunocompromised patients.

## Introduction

Since the onset of the COVID - 19 pandemic, caused by severe acute respiratory syndrome coronavirus 2 (SARS-CoV-2), over seven million people have died worldwide (Worldometer, 2024). In recent years, substantial progress has been made in understanding the epidemiology, pathogenesis, epigenetic influences, hospital-based emergencies, advanced diagnostic technologies, vaccination strategies, and experimental models related to SARS-CoV-2 infection (1–5). However, comparatively less attention has been given to its relationship with the humoral immune response to other human pathogens.

COVID-19 frequently co-occurs with additional infections—including bacterial, viral (especially influenza), fungal, mycobacterial (notably tuberculosis), and latent viral reactivations. These co- and super-infections are generally associated with poorer clinical outcomes, including increased rates of intensive care unit admission, mechanical ventilation, prolonged hospitalization, and higher mortality. Thus, early identification through differential diagnosis and targeted therapeutic interventions—particularly antimicrobial stewardship and combined vaccination strategies—remains critical for optimal patient management.

While some studies report that bacterial co-infection rates in COVID - 19 are lower than those observed in previous influenza pandemics (6), tuberculosis (TB) co-infection continues to pose a major concern. TB–COVID-19 coinfection appears to exacerbate clinical outcomes, with mortality rates remaining higher than those associated with COVID - 19 alone, despite a gradual global decline in TB-related deaths (7). Similarly, respiratory viral co-infections may worsen disease severity and have important prognostic and therapeutic implications (8). Overall, while the current literature provides important insights into co-infections in COVID - 19 patients, many aspects remain insufficiently explored and warrant further investigation (9).

The studies mentioned above are primarily based on the identification of active infections during SARS-CoV-2 infection. However, accurately assessing the full range of current and prior infections in COVID - 19 patients remains technically challenging, given the diversity of potential pathogens involved and the limitations of available diagnostic tools.

The development of mild, moderate, or severe disease in the context of SARS-CoV-2 infection is shaped by complex interactions between genetic and environmental factors, with the immune system playing a central role. Yet, interindividual differences in immune responses and disease severity remain incompletely understood. Among immune mechanisms, antibody responses—especially the production of autoantibodies—have been increasingly recognized as key modulators of the immunopathology observed during acute infection and in post-COVID-19 syndromes (10–15).

Notably, some studies have demonstrated significant clinical improvement in long-COVID patients following two cycles of therapeutic apheresis (16). As a non-specific method for removing circulating antibodies, apheresis led to reduced autoreactivity, supporting the hypothesis that antibody-mediated mechanisms contribute to persistent symptoms. However, the extent to which this procedure affects the overall reactivity of antibodies against microbial pathogens remains unclear. Studies about the complexity of the IgG repertoire in humans, had been discusses as a possible way to uncover neglected functions of antibodies that were not observed due to the complexity and diversity of IgG idiotypes in humans.

Theoretical frameworks have increasingly emphasized that the vast complexity and idiotypic diversity of the human IgG repertoire may obscure fundamental antibody functions, which have remained underappreciated due to the inherent heterogeneity of idiotype expression and the methodological challenges involved in disentangling their immunoregulatory effects (17–19).

In this study, we sought to comprehensively evaluate the anti-pathogen IgG response in COVID - 19 patients, with a focus on identifying differential recognition patterns in individuals with moderate versus severe disease. To achieve this, we employed a high-density peptide microarray comprising over 3,500 unique epitopes derived from 37 viruses, 27 bacteria, 17 parasites, and 8 fungi known to infect humans. Our aim was to identify specific pathogen-related IgG recognition patterns that may distinguish between different clinical severities of COVID - 19.

## Methods

### Samples

Serum samples were obtained from the Central Laboratory Division of the Clinical Hospital, Faculty of Medicine, University of São Paulo (São Paulo, Brazil). All samples were processed by centrifugation to isolate serum and subsequently stored at –20 °C until use.

Participants in the COVID - 19 groups were included based on a confirmed diagnosis of SARS-CoV-2 infection through reverse transcription-polymerase chain reaction (RT-PCR) testing. Individuals over 75 years of age or those with a negative SARS-CoV-2 RT-PCR result were excluded. The final cohort consisted of 79 COVID - 19 patients (39 males and 40 females), stratified into clinical severity groups according to the World Health Organization’s Clinical Management of COVID - 19: Living Guideline (version published on August 18, 2023; WHO).

Patients who were hospitalized with radiological evidence of pneumonia but did not meet criteria for severe or critical disease—and who either received no oxygen therapy or required low-flow oxygen via nasal cannula or face mask—were classified as having moderate COVID - 19 (COVID-Mod; n = 39; mean age: 41.6 ± 6.0 years; 17 males, 22 females). Those requiring high-flow oxygen therapy or non-invasive ventilation due to severe pneumonia were assigned to the severe COVID - 19 group (COVID-Sev; n = 40; mean age: 41.8 ± 6.0 years; 23 males, 17 females). Although mean ages were comparable between groups, the sex distribution differed slightly, which may influence immune response profiles. No patients with critical COVID - 19 were enrolled in this study. All COVID - 19 samples were collected between May and July 2020. Detailed donor information is provided in **Supplementary Table 1**.

Two control groups were included in the study. The first consisted of serum samples from 40 healthy, uninfected individuals (N-exp HC; 17 males, 23 females; mean age: 28.5 ± 2.3 years), collected before the COVID - 19 pandemic (March–July 2019). The second control group comprised a commercially available therapeutic formulation of pooled human IgG intended for intravenous administration (intravenous immunoglobulin; IVIg; Privigen®, CSL Behring), serving as an additional benchmark for polyclonal IgG reactivity.

### Infectious disease epitope microarray

IgG epitope profiling was performed using the PEPperCHIP^®^ Infectious Disease Epitope Microarray (PEPperPRINT GmbH, Heidelberg, Germany), which includes 4,344 linear peptide sequences representing epitopes from 53 viruses, 25 bacteria, 23 parasites, and 1 fungus known to infect humans. The full list of evaluated pathogens, including their acronyms and microorganism categories, is provided in **Supplementary Table 2**. All peptides included on the array are registered in the Immune Epitope Database and Analysis Resource (IEDB; www.iedb.org) and are listed in detail in **Supplementary Table 3**.

As an initial quality control step, microarrays were incubated with only the secondary and control antibodies to detect any nonspecific binding to the immobilized peptides. This background assessment ensured that subsequent sample readings would not be confounded by inherent signal artifacts.

Following quality assurance, human serum samples were diluted at a 1:500 ratio—selected based on prior titration experiments to optimize signal-to-noise ratios—and then applied to the arrays under standardized incubation conditions.

After primary incubation, bound IgG was detected using a fluorescently labeled secondary antibody. Fluorescent signals were visualized and captured using the Innopsys InnoScan 710-IR Microarray Scanner.

To monitor assay performance and slide integrity, hemagglutinin (HA) control peptides positioned along the array perimeter were probed with a dedicated control antibody. These HA signals served as internal positive controls throughout the scanning and analysis workflow.

Image analysis was based on 16-bit grayscale TIFF files, selected for their superior dynamic range compared to 24-bit RGB files used in visual outputs. Signal quantification was performed using an automated pipeline that extracted raw, foreground, and background fluorescence values. For each peptide, median foreground intensities were averaged across technical duplicates. Intra-duplicate variability was evaluated by calculating spot-to-spot deviation.

To maintain data reliability, a maximum deviation threshold of 40% between duplicate spots was enforced. Data points exceeding this variability threshold were excluded from downstream analyses unless individually reviewed and manually classified as either “Artifact” or “Valid.” Intra-slide reproducibility was evaluated by calculating the coefficient of variation (CV%) among technical replicates. Spots with CV > 30% were excluded unless classified as ‘Valid’ based on predefined intensity uniformity and shape criteria.

Corrected median intensities were ranked in descending order to identify the most strongly reactive peptide targets for each sample group. Additionally, spatial signal distribution plots were generated by mapping mean signal intensities from the top-left to bottom-right of the microarray surface, allowing for global assessment of signal uniformity and signal-to-noise ratios.

Final data interpretation incorporated multiple layers of analysis, including quantitative signal ranking, peptide annotation, linear epitope mapping, and visual inspection of microarray scans, to identify dominant IgG-reactive epitopes within each serum group. The results are expressed in arbitrary units (A.U.), calculated as the ratio between the signal obtained from two microarrays: one incubated with only the secondary antibody (background control), and the other with both the sample and the secondary antibody.

To identify epitopes with potentially specific IgG recognition, we applied a threshold defined as the mean signal intensity of the healthy control group plus three standard deviations (mean + 3SD), in accordance with established proteomics standards (20). This stringent cutoff was combined with a minimum signal ratio >2 relative to the comparison group to enhance specificity in identifying pathogens with differential epitope recognition profiles. To further assess the degree of differential IgG reactivity between the COVID-Mod and COVID-Sev groups, group-wise signal intensities were compared using the Mann–Whitney U test followed by Benjamini-Hochberg false discovery rate (FDR) correction, with statistical significance defined as q < 0.05. All p-values and FDR-adjusted q-values for each pathogen are provided in **Supplementary Table 4**.

## Results

### COVID-19 patients produce differential patterns of viral epitope recognition

**Figure 1:**
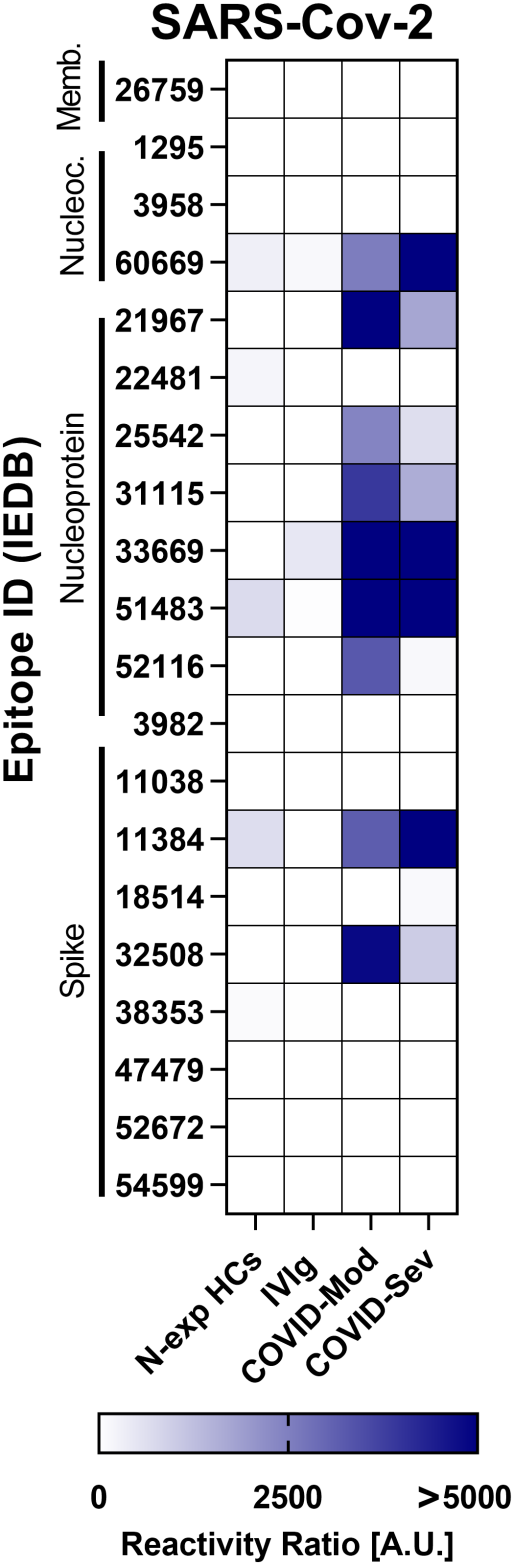
IgG targeting of SARS-CoV-2 epitopes across distinct donor groups. This figure presents data obtained from Infectious Disease Epitope Microarray profiling using serum or purified IgG from four groups: 40 healthy individuals unexposed to SARS-CoV-2 (N-exp HCs), pooled intravenous immunoglobulin (IVIg) derived from thousands of donors, 39 patients with moderate COVID - 19 (COVID-Mod), and 40 patients with severe COVID - 19 (COVID-Sev). Heatmaps display IgG reactivity across the full panel of SARS-CoV-2 epitopes. The source proteins of the SARS-CoV-2 epitopes are indicated along the left side of the heatmap, from top to bottom: membrane glycoprotein, nucleocapsid phosphoprotein, nucleoprotein, and spike glycoprotein. Results are expressed in arbitrary units (A.U.), calculated as the ratio between sample reactivity and background reactivity for each evaluated epitope.

All serum samples were initially assessed for IgG reactivity against 4,344 linear epitopes derived from viral, bacterial, parasitic, and fungal pathogens. As a validation step, we first examined IgG responses to 19 SARS-CoV-2–specific epitopes to confirm expected recognition patterns across groups. As illustrated in **Figure 1**, both COVID-Mod and COVID-Sev patients displayed strong reactivity toward 10 epitopes, while no significant reactivity was observed in the control groups (N-exp HC and IVIg), confirming the specificity of the assay.

**Figure 2:**
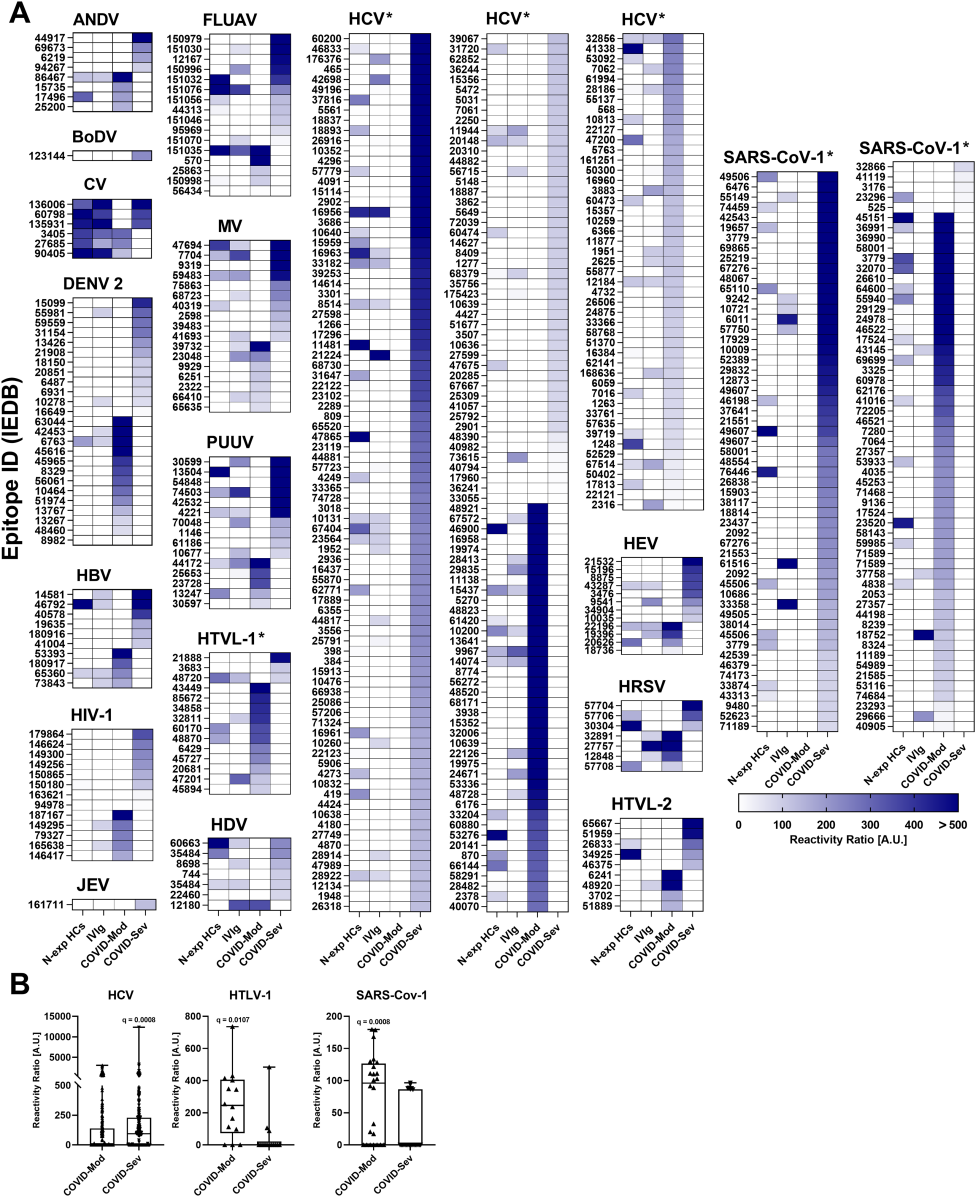
IgG targeting of viral epitopes across distinct donor groups. Data were obtained using the Infectious Disease Epitope Microarray to profile serum or purified IgG from four groups: 40 healthy individuals unexposed to SARS-CoV-2 (N-exp HCs), pooled intravenous immunoglobulin (IVIg) derived from thousands of donors, 39 patients with moderate COVID - 19 (COVID-Mod), and 40 patients with severe COVID - 19 (COVID-Sev). **(A)** Heatmaps depict IgG reactivity against selected viral epitopes showing differential recognition between the COVID-Mod and COVID-Sev groups. The pathogen name is indicated above each heatmap. Within each heatmap, epitopes are ranked by recognition intensity, with those most strongly recognized by IgG from the COVID-Sev group positioned at the top, followed by those preferentially targeted by the COVID-Mod group. Asterisks indicate statistically significant differences between the two groups based on false discovery rate (FDR)-adjusted p-values (*q < 0.05). **(B)** Box-and-whisker plots present detailed IgG reactivity data for all viral epitopes meeting statistical significance (*q < 0.05) in differentiating the COVID-Mod and COVID-Sev groups. Plots show the median, interquartile range, and individual signal intensities for each epitope. Reactivity is expressed in arbitrary units (A.U.), calculated as the ratio of sample reactivity to background reactivity for each evaluated epitope.

Following the confirmation of SARS-CoV-2 epitope reactivity, we next examined IgG responses to epitopes from additional viral pathogens. Distinct recognition patterns emerged between the COVID-Mod and COVID-Sev groups (**Figure 2A**). The Andes virus exhibited eight reactive epitopes, evenly distributed between the two groups. A single epitope from Borna disease virus was uniquely recognized in the COVID-Sev group. Coxsackievirus showed six group-specific epitopes, with three recognized in each group. Dengue virus 2 elicited 25 reactive epitopes—13 specific to COVID-Mod and 12 to COVID-Sev. Hepatitis B virus displayed 10 reactive epitopes, with four unique to COVID-Mod and six to COVID-Sev.

Hepatitis C virus elicited the highest number of reactive epitopes, totaling 219—87 restricted to COVID-Mod and 132 to COVID-Sev. This differential profile was statistically significant, with several epitopes meeting the FDR-adjusted threshold (q < 0.05) (**Figure 2B**). Similarly, for Hepatitis E virus, 12 epitopes were reactive—four exclusive to COVID-Mod and eight to COVID-Sev. HIV - 1 yielded 13 reactive epitopes, five and eight specific to COVID-Mod and COVID-Sev, respectively. HTLV-I showed 14 reactive epitopes, with 11 recognized in COVID-Mod and three in COVID-Sev, a pattern confirmed by statistical analyses (FDR q < 0.05; **Figure 2B**). HTLV-II had nine reactive epitopes—four unique to COVID-Mod and five to COVID-Sev.

The human respiratory syncytial virus was represented by seven reactive epitopes, with four and three specific to COVID-Mod and COVID-Sev, respectively. Influenza A virus displayed 16 epitopes—five in COVID-Mod and 11 in COVID-Sev. Japanese encephalitis virus was recognized by a single epitope in the COVID-Sev group. Measles morbillivirus elicited 17 reactive epitopes—seven restricted to COVID-Mod and ten to COVID-Sev. Puumala virus showed 15 epitopes, with five recognized exclusively by COVID-Mod and ten by COVID-Sev. Lastly, SARS-CoV-1 had 111 reactive epitopes—51 in COVID-Mod and 60 in COVID-Sev. This difference was also supported by statistical analysis (FDR q < 0.05; **Figure 2B**).

Collectively, these findings reveal broad and heterogeneous IgG reactivity toward viral epitopes in COVID - 19 patients, with clear distinctions between moderate and severe disease. These differential patterns suggest that SARS-CoV-2 infection may shape the humoral immune landscape and promote cross-reactivity to unrelated viral antigens.

### COVID-19 patients produce differential patterns of bacterial epitope recognition

**Figure 3:**
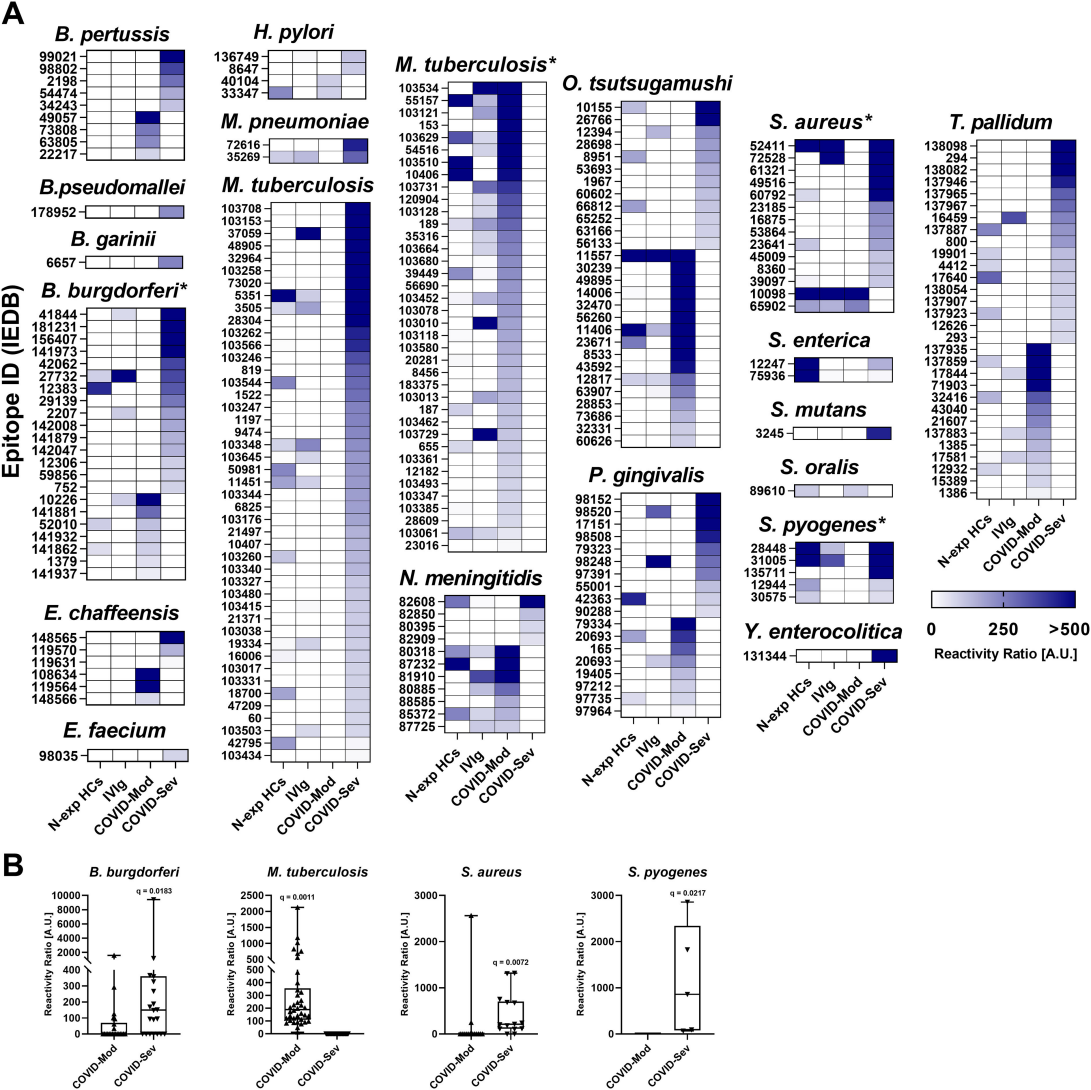
IgG targeting of bacterial epitopes across distinct donor groups. Data were obtained using the Infectious Disease Epitope Microarray to profile serum or purified IgG from four groups: 40 healthy individuals unexposed to SARS-CoV-2 (N-exp HCs), pooled intravenous immunoglobulin (IVIg) derived from thousands of donors, 39 patients with moderate COVID - 19 (COVID-Mod), and 40 patients with severe COVID - 19 (COVID-Sev). **(A)** Heatmaps show IgG reactivity against selected bacterial epitopes exhibiting differential recognition between the COVID-Mod and COVID-Sev groups. The name of each evaluated pathogen is indicated above its corresponding heatmap. Within each heatmap, epitopes are ranked by recognition intensity, with those most strongly recognized by IgG from the COVID-Sev group positioned at the top, followed by those preferentially targeted by the COVID-Mod group. *FDR-adjusted p-values (q < 0.05) indicate statistically significant differences between the COVID-Mod and COVID-Sev groups. **(B)** Detailed IgG reactivity data for the statistically significant (q < 0.05) viral epitopes differentiating the COVID-Mod and COVID-Sev groups. Box-and-whisker plots show the median, interquartile range, and individual signal intensities for each epitope. Results are expressed in arbitrary units (A.U.), calculated as the ratio between sample reactivity and background reactivity for each evaluated epitope.

We further extended our analysis to bacterial epitopes and observed distinct IgG reactivity patterns between the COVID-Mod and COVID-Sev groups (**Figure 3A**). Bordetella pertussis displayed nine differentially recognized epitopes, with four specific to COVID-Mod and five to COVID-Sev. Single epitopes from *Borrelia garinii* and *Burkholderia pseudomallei* were exclusively recognized in the COVID-Sev group. *Borreliella burgdorferi* elicited 22 reactive epitopes—seven in COVID-Mod and 15 in COVID-Sev—a differential profile confirmed by FDR-adjusted statistical analysis (q < 0.05; **Figure 3B**).

*Ehrlichia chaffeensis* exhibited six differentially recognized epitopes, evenly distributed between the groups. Epitopes from *Enterococcus faecium* and *Salmonella enterica* were recognized only in the COVID-Sev group (one and two epitopes, respectively). *Helicobacter pylori* generated four reactive epitopes, with two identified in each group.

*Mycobacterium tuberculosis* emerged as the most extensively recognized bacterium, with 83 differentially reactive epitopes—38 in COVID-Mod and 45 in COVID-Sev. This severity-associated pattern was statistically significant (FDR q < 0.05; **Figure 3B**). Additional epitopes were uniquely recognized in the COVID-Sev group from *Mycoplasma pneumoniae* (two epitopes), *Streptococcus mutans* (one epitope), and *Yersinia enterocolitica* (one epitope), while *Streptococcus oralis* was represented by a single COVID-Mod–specific epitope.

Other species with multiple reactive epitopes included *Neisseria meningitidis* (11 epitopes: seven in COVID-Mod, four in COVID-Sev), *Orientia tsutsugamushi* (22 epitopes: 16 in COVID-Mod, six in COVID-Sev), and *Porphyromonas gingivalis* (18 epitopes: eight in COVID-Mod, ten in COVID-Sev).

*Staphylococcus aureus* displayed 14 reactive epitopes—two specific to COVID-Mod and 12 to COVID-Sev—confirmed as statistically significant (FDR q < 0.05; **Figure 3B**). Similarly, *Streptococcus pyogenes* had five epitopes recognized exclusively in the COVID-Sev group, with the differential profile supported by FDR-adjusted significance (q < 0.05; **Figure 3B**). Finally, *Treponema pallidum* showed 30 reactive epitopes, including 13 unique to COVID-Mod and 17 to COVID-Sev.

These data underscore the complexity of bacterial epitope recognition in COVID - 19, with substantial differences in IgG responses between disease severities. The observed heterogeneity may reflect the modulation of immune recognition pathways triggered by SARS-CoV-2 infection.

### COVID-19 patients produce differential patterns of parasite and fungus epitope recognition

**Figure 4:**
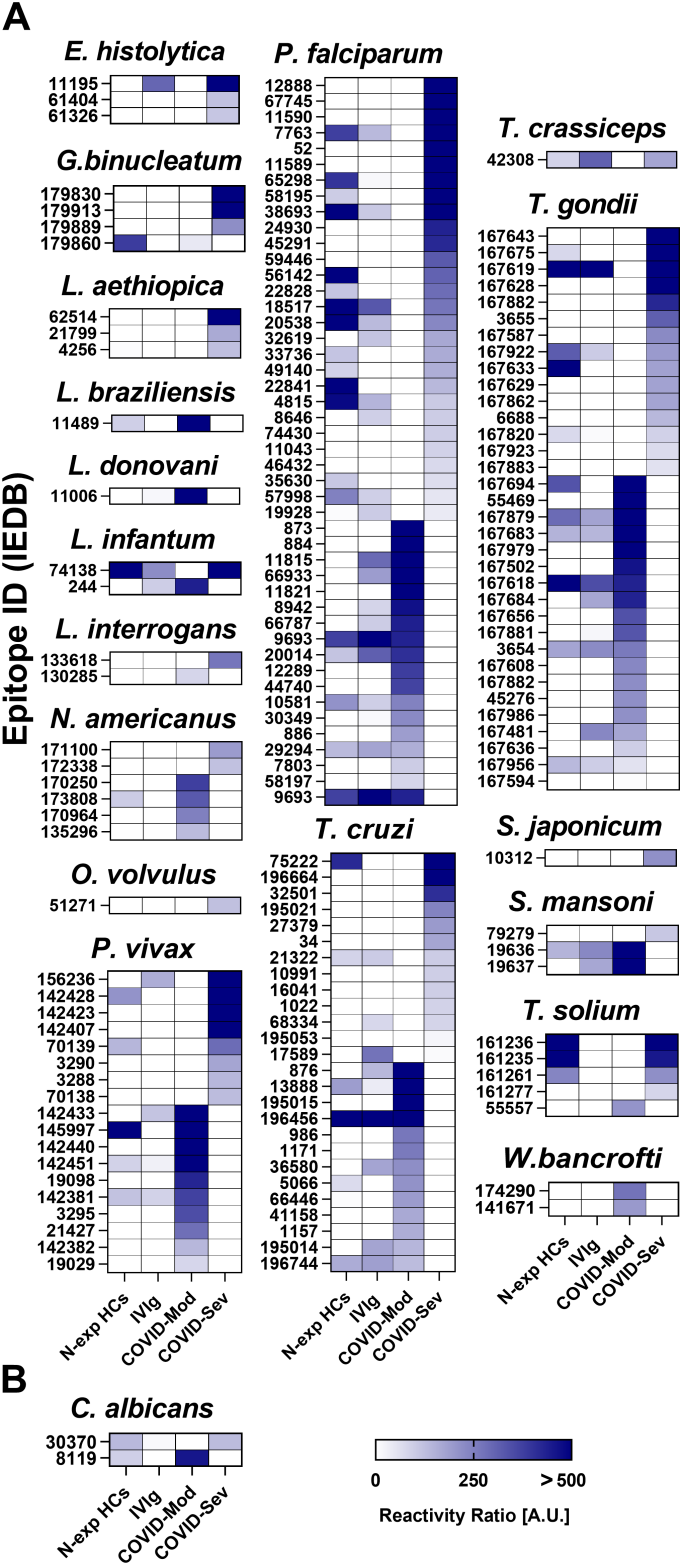
IgG targeting of parasite and fungus epitopes across distinct donor groups. Data were obtained using the Infectious Disease Epitope Microarray to profile serum or purified IgG from four groups: 40 healthy individuals unexposed to SARS-CoV-2 (N-exp HCs), pooled intravenous immunoglobulin (IVIg) derived from thousands of donors, 39 patients with moderate COVID - 19 (COVID-Mod), and 40 patients with severe COVID - 19 (COVID-Sev). **(A)** Heatmaps show IgG reactivity against selected parasite epitopes, and **(B)** heatmaps show IgG reactivity against selected fungus epitopes, both exhibiting differential recognition between the COVID-Mod and COVID-Sev groups. The name of each evaluated pathogen is indicated above its corresponding heatmap. Within each heatmap, epitopes are ranked by recognition intensity, with those most strongly recognized by IgG from the COVID-Sev group positioned at the top, followed by those preferentially targeted by the COVID-Mod group. Results are expressed in arbitrary units (A.U.), calculated as the ratio between sample reactivity and background reactivity for each evaluated epitope.

Finally, we assessed IgG reactivity to parasitic and fungal epitopes and again observed group-specific differences. Among parasites (**Figure 4A**), *Entamoeba histolytica* presented three epitopes recognized exclusively by the COVID-Sev group. *Gnathostoma binucleatum* showed four reactive epitopes—one specific to COVID-Mod and three to COVID-Sev. *Leishmania braziliensis* and *Leishmania donovani* each had one epitope uniquely recognized by COVID-Mod, while *Leishmania aethiopica* had three epitopes recognized only by COVID-Sev. *Leishmania infantum* had two reactive epitopes, one in each COVID group.

*Leptospira interrogans* exhibited two differentially recognized epitopes—one per group. *Necator americanus* displayed six reactive epitopes, four exclusive to COVID-Mod and two to COVID-Sev. A single epitope from *Onchocerca volvulus* was recognized only by COVID-Sev. *Plasmodium falciparum* showed the highest parasite-related reactivity with 46 epitopes—18 unique to COVID-Mod and 28 to COVID-Sev. *Plasmodium vivax* had 18 differentially recognized epitopes, with ten specific to COVID-Mod and eight to COVID-Sev. *Schistosoma japonicum* had one epitope recognized only in COVID-Sev, while *Schistosoma mansoni* displayed three epitopes—two in COVID-Mod and one in COVID-Sev. *Taenia crassiceps* yielded one COVID-Sev–specific epitope, and *Taenia solium* had five—one specific to COVID-Mod and four to COVID-Sev. *Toxoplasma gondii* exhibited 34 reactive epitopes, with 19 recognized only in COVID-Mod and 15 in COVID-Sev. *Trypanosoma cruzi* showed 26 epitopes with a balanced distribution—13 per group. Two epitopes from *Wuchereria bancrofti* were uniquely recognized by COVID-Mod patients. While we observed several differential profiles in IgG recognition of parasitic epitopes, none reached statistical significance according to FDR-adjusted p-values (q < 0.05).

For fungal pathogens (**Figure 4B**), differential IgG recognition was observed only for *Candida albicans*, which exhibited two reactive epitopes—one uniquely recognized by each COVID group; however, these differences did not reach statistical significance.

Together, these results highlight distinct IgG recognition patterns against parasite and fungal epitopes in COVID - 19 patients. The observed group-specific responses may reflect differential immune imprinting shaped by disease severity and prior pathogen exposure.

## Discussion

Our comprehensive IgG epitope profiling across 4,344 pathogen-derived linear peptides demonstrates that SARS-CoV-2 infection induces widespread, severity-dependent alterations in humoral immune responses. These changes shape antibody recognition not just of SARS-CoV-2 epitopes but also of diverse viral, bacterial, parasitic, and fungal antigens, offering a potential new perspective on immune system reprogramming after COVID - 19.

Initially, some technically relevant aspects of the assay used in this study warrant consideration. When testing serum samples against individual epitopes, the resulting data cannot be directly interpreted as evidence of active or past infection. As demonstrated in the analysis of SARS-CoV-2 epitopes, samples from COVID-Mod and COVID-Sev individuals exhibited stronger and more abundant epitope recognition compared to those from non-exposed individuals and the therapeutic IVIg formulation. However, this pattern of recognition cannot be generalized to other epitopes or pathogens assessed in the study. Furthermore, in our results, we only present epitopes that showed differential recognition between the COVID groups, which are the primary focus of our analysis. It is therefore possible that additional epitopes were recognized by the control groups but not by the COVID groups, and these were not included in the current display.

We observed pronounced cross-reactive humoral responses against non–SARS-CoV-2 viruses, notably hepatitis C, SARS-CoV-1, influenza A, dengue, and HTLV, with the strongest responses in patients with severe disease. This may aligns with emerging literature on cross-reactivity and “heterologous immunity,” where immune memory induced by one pathogen influences responses to another (21, 22).

Shrock et al. and Song et al. demonstrated that broader IgG responses and cross-reactivity with conserved spike epitopes are associated with severe COVID - 19, suggesting a combination of pre-existing immunity and *de novo* antibody generation, potentially driven by antigenic mimicry or epitope spreading (22, 23).

Mechanistically, heterologous antibody responses may arise via molecular mimicry, where structural similarities between pathogen antigens—even from bacteria—trigger cross-reactive immune activation (24). Busse et al. demonstrated *in vitro* that sensitization with bacterial peptides homologous to SARS-CoV-2 induces cross-reactive T cell immunity (25). Our observation of IgG reactivity to peptides from commensals such as *M. tuberculosis*, *S. aureus*, and *P. gingivalis* suggests humoral extensions of this heterologous response. Echoing this, emerging data indicate that microbiome-derived peptides can shape early SARS-CoV-2 immune responses (26).

Differential epitope recognition between moderate and severe COVID - 19 cases, especially for *HCV* and *M. tuberculosis*, indicates that severe infection may be associated with more extensive antibody spreading or activation. This is consistent with reports that severe disease induces epitope breadth expansion due to systemic inflammation and dysregulated B cell responses (27). Such dysregulated responses have also been implicated in autoantibody development in COVID - 19 (28, 29), and our results suggest possible collateral reactivity to microbial and self-antigens. While antibody-dependent enhancement (ADE) remains rare in COVID - 19 (30), identification of cross-reactive epitopes warrants functional investigation for neutralizing versus pathogenic roles.

Beyond observational patterns, growing evidence indicates that the IgG repertoire may play an active immunoregulatory role. Qualitative differences in IgG extend beyond antigen specificity and can directly influence immune cell function. Several studies have shown that polyclonal IgG molecules are capable of modulating activation thresholds, cytokine secretion profiles, and the phenotypic differentiation of both T and B cells—independently of classical Fc receptor engagement—in diverse pathological contexts, including allergies (31–36), atopic dermatitis (37–40), and viral infections (41, 42). Furthermore, in an innovative approach, Nahm et al. demonstrated that the *in vivo* administration of polyclonal IgG obtained from patients with atopic dermatitis can exert immunomodulatory effects on peripheral T cells, leading to clinical improvement in these patients (43–47). Together, these *in vitro* and *in vivo* observations—although not obtained in the specific context of viral infection—suggest that distinct sets of IgG idiotypes can effectively modulate immune responses, with potential clinical implications. In the present study, we observed marked differences in IgG recognition of several epitopes. This differential reactivity profile is likely associated with distinct sets of IgG idiotypes between moderate and severe COVID - 19 cases. Although our analysis did not assess these variations longitudinally, we propose that, at the time of each clinical manifestation, the specific IgG idiotype repertoire present in moderate or severe COVID - 19 could theoretically involve immunomodulatory effects on self-immunity.

Recent proteomic analyses have demonstrated that IgG preparations from individuals with varying degrees of SARS-CoV-2 disease severity can elicit distinct patterns of interaction with multiple human tissues and diverse peripheral blood cell types (15, 48).

Mechanistic studies have also demonstrated that IgG from individuals with varying degrees of SARS-CoV-2 disease severity can function as a modulatory molecule, influencing the activity of peripheral mucosal-associated invariant T (MAIT) cells (49). These findings support the hypothesis that the SARS-CoV-2–induced shifts in the IgG repertoire we observed—particularly in patients with severe disease—may not only reflect downstream immune activation but may also contribute actively to shaping immune responses through direct modulation of lymphocyte function.

Clinically, severity-associated epitope signatures may serve as a basis for the development of future biomarkers. For example, elevated recognition of hepatitis C or SARS-CoV-1 peptides in severe patients may reflect broader B cell activation or predisposing immune dysregulation. Epitope-based seroprofiling could serve as a tool to stratify patients and inform prognosis.

Our study has several limitations. First, we did not evaluate the functional properties of the identified antibodies—such as their neutralizing capacity or Fc-mediated effector functions—which are essential for determining clinical relevance. Second, comprehensive clinical histories of the participants, including prior infections, vaccination status, and sex- and age-related immune variables, were not fully documented. These factors may act as potential confounders and should be carefully considered in future stratified analyses.

Third, the cross-sectional study design limits temporal interpretation; it remains unclear whether the observed epitope reactivities predated or resulted from SARS-CoV-2 infection. Finally, the absence of an independent validation cohort restricts the generalizability of our findings; future studies should address this by including longitudinal sampling and external validation across diverse populations.

Despite these limitations, our findings offer important insights. They underscore the complexity of post-COVID immune landscapes and may suggest potential roles for epitope spreading and molecular mimicry in modulating humoral responses. These observations may inform novel strategies for biomarker development, vaccine design, and patient stratification. A deeper understanding of how SARS-CoV-2 infection reshapes the IgG repertoire—including cross-reactivity to antigens from unrelated pathogens—may theoretically contribute to future pandemic preparedness and the management of post-infectious syndromes. Although preliminary, the severity-associated epitope signatures described here may ultimately support personalized immunomonitoring approaches for co-infected or immunocompromised individuals, pending further validation.

## Data Availability

The original contributions presented in the study are included in the article/**Supplementary Material**. Further inquiries can be directed to the corresponding author.

## References

[1] Zhou P Yang XL Wang XG Hu B Zhang L Zhang W. A pneumonia outbreak associated with a new coronavirus of probable bat origin. Nature. (2020) 579:270–3. doi: 10.1038/s41586-020-2012-7, PMID: 32015507 PMC7095418

[2] Contini C Rotondo JC Perna B Guarino M De Giorgio R. Special issue: advances in SARS-coV-2 infection. Microorganisms. (2023) 11(4):1048. doi: 10.3390/microorganisms11041048, PMID: 37110471 PMC10145712

[3] Li X Mi Z Liu Z Rong P. SARS-CoV-2: pathogenesis, therapeutics, variants, and vaccines. Front Microbiol. (2024) 15:1334152. doi: 10.3389/fmicb.2024.1334152, PMID: 38939189 PMC11208693

[4] Chung YS Lam CY Tan PH Tsang HF Wong SC. Comprehensive review of COVID-19: epidemiology, pathogenesis, advancement in diagnostic and detection techniques, and post-pandemic treatment strategies. Int J Mol Sci. (2024) 25(15):8155. doi: 10.3390/ijms25158155, PMID: 39125722 PMC11312261

[5] Rotondo JC Martini F Maritati M Caselli E Gallenga CE Guarino M. Advanced molecular and immunological diagnostic methods to detect SARS-coV-2 infection. Microorganisms. (2022) 10(6):1193. doi: 10.3390/microorganisms10061193, PMID: 35744711 PMC9231257

[6] Lansbury L Lim B Baskaran V Lim WS. Co-infections in people with COVID-19: a systematic review and meta-analysis. J Infect. (2020) 81:266–75. doi: 10.1016/j.jinf.2020.05.046, PMID: 32473235 PMC7255350

[7] Wang Q Cao Y Liu X Fu Y Zhang J Zhang Y. Systematic review and meta-analysis of Tuberculosis and COVID-19 Co-infection: Prevalence, fatality, and treatment considerations. PloS Negl Trop Dis. (2024) 18:e0012136. doi: 10.1371/journal.pntd.0012136, PMID: 38739637 PMC11090343

[8] Krumbein H Kümmel LS Fragkou PC Thölken C Hünerbein BL Reiter R. Respiratory viral co-infections in patients with COVID-19 and associated outcomes: A systematic review and meta-analysis. Rev Med Virol. (2023) 33:e2365. doi: 10.1002/rmv.2365, PMID: 35686619 PMC9347814

[9] Kim JYH Ragusa M Tortosa F Torres A Gresh L Méndez-Rico JA. Viral reactivations and co-infections in COVID-19 patients: a systematic review. BMC Infect Dis. (2023) 23:259. doi: 10.1186/s12879-023-08117-y, PMID: 37101275 PMC10131452

[10] Notarte KI Carandang THDC Velasco JV Pastrana A Ver AT Manalo GN. Autoantibodies in COVID-19 survivors with post-COVID symptoms: a systematic review. Front Immunol. (2024) 15:1428645. doi: 10.3389/fimmu.2024.1428645, PMID: 39035011 PMC11257835

[11] Delle Fave RF Polisini G Giglioni G Parlavecchio A Dell'Atti L Galosi AB. COVID-19 and male fertility: Taking stock of one year after the outbreak began. Arch Ital Urol Androl. (2021) 93:115–9. doi: 10.4081/aiua.2021.1.115, PMID: 33754623

[12] Van Regemorter E Zorzi G Scohy A Gruson D Morelle J. Impact of the COVID-19 pandemic on temporal trends of biological indicators of autoimmunity. J Transl Autoimmun. (2023) 7:100222. doi: 10.1016/j.jtauto.2023.100222, PMID: 38074080 PMC10704428

[13] Klein J Wood J Jaycox JR Dhodapkar RM Lu P Gehlhausen JR. Distinguishing features of long COVID identified through immune profiling. Nature. (2023) 623:139–48. doi: 10.1038/s41586-023-06651-y, PMID: 37748514 PMC10620090

[14] Wang EY Mao T Klein J Dai Y Huck JD Jaycox JR. Diverse functional autoantibodies in patients with COVID-19. Nature. (2021) 595:283–8. doi: 10.1038/s41586-021-03631-y, PMID: 34010947 PMC13130511

[15] MaChado NR Fagundes BO do Nascimento LA Bergamasco IS Sgnotto FDR Fernandes IG. Deciphering the igG idiotype network through proteomic analysis of potential targets in SARS-coV-2-induced immune responses. Immunology. (2025) 175(2):226–39. doi: 10.1111/imm.13919, PMID: 40077865

[16] Achleitner M Steenblock C Dänhardt J Jarzebska N Kardashi R Kanczkowski W. Clinical improvement of Long-COVID is associated with reduction in autoantibodies, lipids, and inflammation following therapeutic apheresis. Mol Psychiatry. (2023) 28:2872–7. doi: 10.1038/s41380-023-02084-1, PMID: 37131073 PMC10152027

[17] Victor JR. Do different IgG repertoires play a role in B- and T-cell functional modulation during ontogeny? The "hooks without bait" theory. Immunol Cell Biol. (2020) 98:540–8. doi: 10.1111/imcb.12335, PMID: 32342552

[18] Victor JR. Allergen-specific IgG as a mediator of allergy inhibition: Lessons from mother to child. Hum Vaccines Immunotherapeutics. (2017) 13:507–13. doi: 10.1080/21645515.2016.1244592, PMID: 27808600 PMC5360138

[19] Victor JR. Influence of maternal immunization with allergens on the thymic maturation of lymphocytes with regulatory potential in children: a broad field for further exploration. J Immunol Res. (2014) 2014:780386. doi: 10.1155/2014/780386, PMID: 25009823 PMC4070472

[20] Pugh S Fosdick BK Nehring M Gallichotte EN VandeWoude S Wilson A. Estimating cutoff values for diagnostic tests to achieve target specificity using extreme value theory. BMC Med Res Methodol. (2024) 24:30. doi: 10.1186/s12874-023-02139-5, PMID: 38331732 PMC10851584

[21] Murray SM Ansari AM Frater J Klenerman P Dunachie S Barnes E. The impact of pre-existing cross-reactive immunity on SARS-CoV-2 infection and vaccine responses. Nat Rev Immunol. (2023) 23:304–16. doi: 10.1038/s41577-022-00809-x, PMID: 36539527 PMC9765363

[22] Geanes ES LeMaster C Fraley ER Khanal S McLennan R Grundberg E. Cross-reactive antibodies elicited to conserved epitopes on SARS-CoV-2 spike protein after infection and vaccination. Sci Rep. (2022) 12:6496. doi: 10.1038/s41598-022-10230-y, PMID: 35444221 PMC9019795

[23] Shrock E Fujimura E Kula T Timms RT Lee IH Leng Y. Viral epitope profiling of COVID-19 patients reveals cross-reactivity and correlates of severity. Science. (2020) 370(6520):eabd4250. doi: 10.1126/science.abd4250, PMID: 32994364 PMC7857405

[24] Ding Z Wei X Pan H Shi H Shi Y. Unveiling the intricacies of COVID-19: Autoimmunity, multi-organ manifestations and the role of autoantibodies. Scand J Immunol. (2024) 99:e13344. doi: 10.1111/sji.13344, PMID: 39007954

[25] Eggenhuizen PJ Ng BH Chang J Cheong RMY Yellapragada A Wong WY. Heterologous immunity between SARS-coV-2 and pathogenic bacteria. Front Immunol. (2022) 13:821595. doi: 10.3389/fimmu.2022.821595, PMID: 35154139 PMC8829141

[26] Gaebler C Wang Z Lorenzi JCC Muecksch F Finkin S Tokuyama M. Evolution of antibody immunity to SARS-CoV-2. Nature. (2021) 591:639–44. doi: 10.1038/s41586-021-03207-w, PMID: 33461210 PMC8221082

[27] Woodruff MC Ramonell RP Nguyen DC Cashman KS Saini AS Haddad NS. Extrafollicular B cell responses correlate with neutralizing antibodies and morbidity in COVID-19. Nat Immunol. (2020) 21:1506–16. doi: 10.1038/s41590-020-00814-z, PMID: 33028979 PMC7739702

[28] Zuo Y Estes SK Ali RA Gandhi AA Yalavarthi S Shi H. Prothrombotic autoantibodies in serum from patients hospitalized with COVID-19. Sci Transl Med. (2020) 12(570):eabd3876. doi: 10.1126/scitranslmed.abd3876, PMID: 33139519 PMC7724273

[29] Ehrenfeld M Tincani A Andreoli L Cattalini M Greenbaum A Kanduc D. Covid-19 and autoimmunity. Autoimmun Rev. (2020) 19:102597. doi: 10.1016/j.autrev.2020.102597, PMID: 32535093 PMC7289100

[30] Lee WS Wheatley AK Kent SJ DeKosky BJ. Antibody-dependent enhancement and SARS-CoV-2 vaccines and therapies. Nat Microbiol. (2020) 5:1185–91. doi: 10.1038/s41564-020-00789-5, PMID: 32908214 PMC12103240

[31] Cunha FRM Fagundes BO MaChado NR França CN Victor JR. IgG from individuals without atopy arising as mediators of a nonatopic profile in human peripheral CD4+ T cells. Ann Allergy Asthma Immunol. (2024) 132:770–2. doi: 10.1016/j.anai.2024.03.014, PMID: 38521342

[32] de-Apoena Reche DT MaChado NR Fagundes BO Bergamasco IS de Sousa TR do Nascimento LA. IgG from Dermatophagoides pteronyssinus (Der p)-atopic individuals modulates non-atopic thymic B cell phenotype (alfa-4/beta-7) and cytokine production (IFN-γ, IL-9, and IL-10) with direct membrane interaction. Sci Rep. (2024) 14:7274. doi: 10.1038/s41598-024-57950-x, PMID: 38538762 PMC10973508

[33] Sousa TRD Sgnotto FDR Fagundes BO Duarte AJDS Victor JR. Non-atopic neonatal thymic innate lymphoid cell subsets (ILC1, ILC2, and ILC3) identification and the modulatory effect of igG from dermatophagoides pteronyssinus (Derp)-atopic individuals. Front Allergy. (2021) 28:650235. doi: 10.3389/falgy.2021.650235, PMID: 35387031 PMC8974683

[34] Rodrigues de Sousa T da Ressureição Sgnotto F Oliveira Fagundes B Souza Santos L da Silva Duarte AJ Victor JR. IgG from atopic individuals can mediate non-atopic infant thymic and adult peripheral CD8. Eur Ann Allergy Clin Immunol. (2021) 53:161–7. doi: 10.23822/EurAnnACI.1764-1489.157, PMID: 32548997

[35] Inoue AHS Lira AAL de-Oliveira MG de Sousa TR Sgnotto FDR Duarte AJDS. The potential of igG to induce murine and human thymic maturation of IL-10+ B cells (B10) revealed in a pilot study. Cells. (2020) 9(10):2239. doi: 10.3390/cells9102239, PMID: 33027887 PMC7600151

[36] Santos LS Sgnotto FDR Inoue AHS Padreca AF Menghini RP Duarte AJDS. IgG from non-atopic individuals induces *in vitro* IFN-γ and IL-10 production by human intra-thymic γδT cells: A comparison with atopic igG and IVIg. Arch Immunol Ther Exp (Warsz). (2019) 67(4):263–70. doi: 10.1007/s00005-019-00545-6, PMID: 31087106

[37] Fagundes BO de Sousa TR Nascimento A Fernandes LA Sgnotto FDR Orfali RL. IgG from adult atopic dermatitis (AD) patients induces nonatopic neonatal thymic gamma-delta T cells (γδT) to acquire IL-22/IL-17 secretion profile with skin-homing properties and epigenetic implications mediated by miRNA. Int J Mol Sci. (2022) 23(12):6872. doi: 10.3390/ijms23126872, PMID: 35743310 PMC9224404

[38] de Sousa TR Fagundes BO Nascimento A Fernandes LA Sgnotto FDR Orfali RL. IgG from adult atopic dermatitis (AD) patients induces thymic IL-22 production and CLA expression on CD4+ T cells: possible epigenetic implications mediated by miRNA. Int J Mol Sci. (2022) 23(12):6867. doi: 10.3390/ijms23126867, PMID: 35743308 PMC9224968

[39] Santos LS Sgnotto FDR Sousa TR Orfali RL Aoki V Duarte AJDS. IgG from atopic dermatitis patients induces non-atopic infant thymic invariant natural killer T (iNKT) cells to produce IL-4, IL-17, and IL-10. Int J Dermatol. (2019) 59(3):359–64. doi: 10.1111/ijd.14688, PMID: 31631342

[40] Sgnotto FDR de Oliveira MG Lira AAL Inoue AHS Titz TO Orfali RL. IgG from atopic dermatitis patients induces IL-17 and IL-10 production in infant intrathymic TCD4 and TCD8 cells. Int J Dermatol. (2018) 57:434–40. doi: 10.1111/ijd.13907, PMID: 29355930

[41] MaChado NR Fagundes BO Oliveira ACP Nukui Y Casseb J Cunha FR. Differential modulation of IL-4, IL-10, IL-17, and IFN-γ production mediated by IgG from Human T-lymphotropic virus-1 (HTLV-1) infected patients on healthy peripheral T (CD4+, CD8+, and γδ) and B cells. Front Med. (2023) 10:1239706. doi: 10.3389/fmed.2023.1239706, PMID: 37711742 PMC10498471

[42] da Ressureição Sgnotto F Souza Santos L Rodrigues de Sousa T Feitosa de Lima J Mara da Silva Oliveira L Saeed Sanabani S. IgG from HIV-1-exposed seronegative and HIV-1-infected subjects differently modulates IFN-γ Production by thymic T and B cells. J Acquir Immune Defic Syndr. (2019) 82:e56–60. doi: 10.1097/QAI.0000000000002182, PMID: 31714433

[43] Nahm DH Kim ME Cho SM. Effects of intramuscular injection of autologous immunoglobulin on clinical severity and serum igE concentration in patients with atopic dermatitis. Dermatology. (2015) 231:145–51. doi: 10.1159/000431173, PMID: 26112673

[44] Nahm DH Ahn A Kim ME Cho SM Park MJ. Autologous immunoglobulin therapy in patients with severe recalcitrant atopic dermatitis: long-term changes of clinical severity and laboratory parameters. Allergy Asthma Immunol Res. (2016) 8:375–82. doi: 10.4168/aair.2016.8.4.375, PMID: 27126731 PMC4853515

[45] Cho SM Kim ME Kwon B Nahm DH. Immunomodulatory effects induced by intramuscular administration of autologous total immunoglobulin G in patients with atopic dermatitis. Int Immunopharmacol. (2017) 52:1–6. doi: 10.1016/j.intimp.2017.08.020, PMID: 28846886

[46] Nahm DH Ye YM Shin YS Park HS Kim ME Kwon B. Efficacy, safety, and immunomodulatory effect of the intramuscular administration of autologous total immunoglobulin G for atopic dermatitis: A randomized clinical trial. Allergy Asthma Immunol Res. (2020) 12:949–63. doi: 10.4168/aair.2020.12.6.949, PMID: 32935488 PMC7492515

[47] Nahm DH Kim ME Kwon B Kim JS Park B. Intramuscular injection of autologous serum in adolescent and adult patients with atopic dermatitis: A preliminary randomized clinical trial. Yonsei Med J. (2023) 64:423–32. doi: 10.3349/ymj.2022.0559, PMID: 37365736 PMC10307681

[48] MaChado NR do Nascimento LA Fagundes BO Borges JVDS Sgnotto FDR Bergamasco IS. Proteomic profiling of human peripheral blood cell targets of igG induced by SARS-coV-2: insights into vaccine safety. Vaccines (Basel). (2025) 13(7):694. doi: 10.3390/vaccines13070694, PMID: 40733671 PMC12299517

[49] Rakanidis MaChado N Fagundes BO Fernandes IG Terra De Apoena Reche D Sato MN Victor JR. IgG from patients with mild or severe COVID−19 reduces the frequency and modulates the function of peripheral mucosal-associated invariant T cells in PBMCs from healthy individuals. BioMed Rep. (2023) 19:95. doi: 10.3892/br.2023.1677, PMID: 37901873 PMC10603374

